# The Manufacturing Conditions for the Direct and Reproducible Formation of Electrospun PCL/Gelatine 3D Structures for Tissue Regeneration

**DOI:** 10.3390/nano13243107

**Published:** 2023-12-09

**Authors:** Chloe Jayne Howard, Aumrita Paul, Justin Duruanyanwu, Kenza Sackho, Paola Campagnolo, Vlad Stolojan

**Affiliations:** 1 Advanced Technology Institute, School of Computer Science and Electronic Engineering, University of Surrey, Guildford GU2 7XH, UK; ch01148@surrey.ac.uk (C.J.H.); ap02390@surrey.ac.uk (A.P.); 2 Department of Biochemical Sciences, University of Surrey, Guildford GU2 7XH, UK; j.druanyanwu@surrey.ac.uk (J.D.); k.sackho@surrey.ac.uk (K.S.); p.campagnolo@surrey.ac.uk (P.C.)

**Keywords:** electrospinning, sponge scaffolds, PCL, concentration, humidity, conductivity, cell viability, tissue engineering

## Abstract

Electrospinning is a versatile technique for fabricating nanofibrous scaffolds for tissue engineering applications. However, the direct formation of 3D sponges through electrospinning has previously not been reproducible. We used a Taguchi experimental design approach to optimise the electrospinning parameters for forming PCL and PCL/gelatine 3D sponges. The following parameters were investigated to improve sponge formation: solution concentration, humidity, and solution conductivity. Pure PCL sponges were achievable. However, a much fluffier sponge formed by increasing the solution conductivity with gelatine. The optimal conditions for sponge formation 24 *w*/*v*% 80:20 PCL:gelatine on aluminium foil at ≥70% humidity, 15 cm, 22 kV and 1500 µL/h. The resulting sponge had a highly porous structure with a fibre diameter of ~1 µm. They also supported significantly higher cell viability than 2D electrospun mats, dropcast films of the same material and even the TCP positive control. Our study demonstrates that the direct formation of PCL/gelatine 3D sponges through electrospinning is feasible and promising for tissue engineering applications. The sponges have a highly porous structure and support cell viability, which are essential properties for tissue engineering scaffolds. Further studies are needed to optimise the manufacturing process and evaluate the sponges’ long-term performance in vivo.

## 1. Introduction

Tissue engineering has the potential to improve the lives of many suffering from disease or injury [1,2]. It provides the possibility of restoring lost or damaged tissue, reducing scarring, treating disease and even the possibility of creating organs [2]. The engineering behind tissue regeneration relies on creating an environment that mimics the targeted tissue and is biocompatible, increasing mechanical support to cells and allowing for the exchange of bioactive molecules [3]. Currently, tissue repair is based on the use of allogeneic (from a donor of the same species) or autologous (from the patient’s tissue) grafts. However, these options are limited by issues with transplant rejection and tissue availability [4]. 

The development of synthetic scaffolds to support the growth and organisation of new tissue [5] provides a potential route to address these challenges. Several biocompatible materials, such as polymers, metals and ceramics, have been tested for tissue engineering [6,7] with synthetic polymeric nanofiber scaffolds providing advantages such as (a) a high surface area for cell attachment; (b) high porosity for bioactive exchange; (c) a biomimetic structure that mimics the extracellular matrix (ECM); and (d) the ability to be tailored to specific cell types and applications [7].

Among fabrication techniques, electrospinning stands out due to its simplicity, scalability, low cost and adaptability. This technique has been successful in producing grafts for the regeneration of a broad range of tissues, such as skin [8,9], bone [4,10,11,12], cartilage [13,14,15,16] and muscle [17,18], by modulating the parameters of the graft production in accordance to the requirements of the final product. For example, muscle fibres are aligned in the direction of contraction, which can be mimicked by producing aligned electrospun fibres, guiding the orientation of the seeded cells [18,19]. It is important to note that electrospun scaffolds are generally two-dimensional, limiting their application in tissue engineering [20] as they offer limited options for cell penetration and do not provide sufficient volume to replace tissue loss [21].

A truly three-dimensional scaffold that leverages the properties of electrospun mats is required to further the development of tissue repair. Researchers have attempted to build 3D structures from electrospun fibres by cutting and freeze-drying them (in the presence of a binder), thus obtaining sponge structures with a very high porosity (99.6%) and very low density (<3 mg·cm^−3^) [22,23]. However, this solution requires additional processing and toxic solvents and can lead to a loss of mechanical integrity [20,21]. 

The direct formation of 3D electrospun structures has been reported previously from PAN, cellulose acetate, polystyrene and zein; however, they are not easily reproduced, and their taxonomy varies [19,24,25,26].

The formation of these sponges has been attributed to several factors, with the most common explanation being that it is due to the electrical conductivity of the fibres. The fibres may be attracted to the needle tip due to their higher relative conductivity [26] or repel each other [19]. This effect has also been attributed to residual charge, where polymers with low conductivity and high surface resistivity would build up more residual charge and lead to the repulsion of fibres from each, leading to loftiness [24]. 

Humidity has also been shown to play a significant role in sponge formation because water has antisolvent properties for all polymer solutions that form direct electrospun sponges, causing a more rapid solidification and increased fibre mechanical strength [24,25].

In addition, the current sponge materials have limitations due to the release of toxic breakdown products and poor biodegradability [19,24,25,26].

Here, we aim to identify the reproducible conditions for the direct electrospinning of a sponge from polycaprolactone (PCL), a polymer chosen for its biocompatibility, biodegradability, and previous success as a 2D electrospun tissue scaffold [11,27,28,29,30].

However, PCL degrades very slowly, typically over 6-12 months, which may represent a limitation in some tissue engineering applications. Gelatine is a fast-degrading natural polymer [11], which has been shown to hasten overall degradation when co-electrospun with PCL [31]. On the other hand, adding PCL to gelatine improves the mechanical properties [32]. 

In this study, we demonstrate the successful and reproducible direct electrospinning of PCL and PCL/gelatine 3D sponges, which has not previously been completed, and show that the PCL/gelatine sponges have over double the cell viability after 7 days compared to 2D electrospun mats from the same material and a dropcast film of the same material.

As the PCL sponge manufacturing results inform the PCL/gelatine manufacturing, we organised the paper to first analyse and discuss the PCL sponge results, leading to the PCL/gelatine results and discussion.

## 2. Materials and Methods

### 2.1. Materials

PCL, average molecular weight ∼ 80,000, and gelatine from porcine skin were purchased from Merck (Gillingham, UK). Acetic acid (AA), ≥95% and formic acid (FA), ≥99.8% were purchased from Thermo Scientific Chemicals (Leicestershire, UK). Phosphate buffered saline (PBS) was purchased from Merck (Gillingham, UK). A Normal Human Dermal Fibroblasts (NDHF) cell line and Fibroblast growth medium 2 (FGM2) were purchased from Promocell (Heidelberg, Germany). The AlamarBlue™ Cell Viability Reagent, 4′,6-Diamidino-2-Phenylindole, Dihydrochloride (DAPI), and CellTracker™ Red CMTPX Dye were all purchased from Fisher Scientific. Trypsin-EDTA (0.25%), phenol red was purchased from Gibco™ (Leicestershire, UK).

### 2.2. Method: Electrospinning Solution Preparation and Instrumentation

PCL solutions were prepared at 14, 18, 22, 26 and 28 *w*/*v*% in a 1:1 AA:FA ratio. The solution was stirred for 3 h at room temperature and then placed in a 5 mL syringe. 

Gelatine was combined with PCL with the ratios of PCL:gelatine 60:40 and 80:20, in a 1:1 AA:FA solution. The combined PCL:gelatine concentrations were 22 *w*/*v*% and 24 *w*/*v*%. As PCL and gelatine form an solution [33], the solution was electrospun immediately to prevent separation.

The electrospinning apparatus was housed in a bespoke fume hood at ambient temperature and humidity (recorded using a RS Hygrometer) and consisted of a syringe pump, a syringe with a stainless-steel needle and a flat collector (40 cm × 40 cm).

The syringe was fitted into a syringe pump, and the solution was pumped through a stainless steel 21-gauge needle. The feed rate was controlled using a Chemyx Syringe Pump driver. A high potential field was generated using a high-voltage DC power supply (Glassman FJ60, Gloucester, UK). The feed rate, high-voltage and needle-to-collector distance were study parameters, and each was varied at three levels between 500 µL/h and 2,000 µL/h, 12 kV and 20 kV and 10 cm and 20 cm, respectively. The collector consisted of a flat stainless-steel surface covered with silicone release paper (Amazon, London, UK) or Aluminium foil (RS Components, Northants, UK).

The room humidity (RH) in the electrospinning enclosure was increased >60%RH by evaporating water on a hot plate, when the ambient humidity was <40%RH.

### 2.3. Method: Taguchi Design of Experiments

Given the high number of electrospinning experimental variables, we reduce the number of experiments through the Taguchi Desing of Experiments method. The Taguchi method is used to improve the quality of a product or process by minimising variability and maximising output [34,35], by looking at pairs of parameters, and is very useful in finding a manufacturing envelope. We define a manufacturing quality score to identify the sponge manufacturing conditions with the Taguchi method (Table 1). This is an empirical score based on observations during fibre deposition.

As there were three levels for each of the variable parameters defined in Section 2.2, we used an L_9_ Taguchi orthogonal array define experimental conditions for each run (Table 2), where humidity and room temperature were treated as noise. This orthogonal array comes from the projection of the three parameter, three level experiment, which would traditionally require 3^3^ experiments, onto pairs of parameters on axes, reducing the number of unique points to nine [36]. These unique points are given in the L_9_ table. Our L_9_ table was generated using Minitab^®^ 21, which was also used to statistically analyse the results.

### 2.4. Method: Nanofibre and Sponge Characterisation

Nanofibre diameter was measured using Scanning Electron Microscopy (SEM) on a field-emission Tescan Mira II. Fibres were collected after deposition, placed flat onto a double-sided sticky carbon tab (Agar Scientific, Stansted, UK) and placed on 12.5 mm diameter aluminium pin stub (Agar Scientific, Stansted, UK). The samples were sputter-coated with Au (6 nm, Quorum Technologies Q150T sputter coater, Lewes, UK) to prevent charging during imaging. For each sample, >100 nanofibres (10 images at 15 µm view field) were measured using ImageJ and the average diameter and standard deviation were calculated.

The density and porosity of the sponges were calculated as a percentage of the weight of the sponge to a solid polymer (PCL or PCL/gelatine) of the same volume (Equation (1) [37]). To measure the volume of the sponge accurately, the sponges were submerged in water and then fitted into a cylindrical mould (a 5 mL syringe sleeve); this may have led to some compression; however, it allows for a more accurate calculation of the volume. Once frozen, the sleeve was removed, and the sponge was cut to ~1 cm height. The frozen sponge was then freeze-dried in a Lablyo Mini freezer dryer for 48 h. The outer dimensions of the sponge were measured to ~5% tolerance using a micrometre:

(1) $$\varphi =1-\frac{m}{\rho_{bulk}\cdot V}\times 100\%$$

Fourier transform infrared spectroscopy (FTIR) (Agilent Varian 660, Didcot, UK) was used to confirm the presence of gelatine in the PCL/gelatine sponge at a resolution of 2 cm^−1^ [33]. 

The hydrophilicity was measured using Krüss Advance Scientific. The sessile drop technique with deionized water was used to find the initial mean contact angle and to measure the rate of change in the contact angle in time. A lower initial mean contact angle and an increased rate of angle reduction indicate a more hydrophilic sponge.

### 2.5. Method: Cytocompatibility Study

The PCL/gelatine sponge was compared to a 2D electrospun PCL/gelatine nanofibre mat, and a drop-cast film made from the same PCL/gelatine solution used for electrospinning. In addition, we also compared the sponge against a positive control of plasma-treated polystyrene (PTP) fabricated from polystyrene (Merck UK) and oxygen–plasma treated at 20 W for 20 s for hydrophilicity. 

All 2D substrates had a cross-sectional area of 0.25 cm^2^; the 3D sponges also had a base cross-sectional area of 0.25 cm^2^ and a height of approximately 0.5 cm. 

The samples were placed into a 24-well plate and sterilised under UV light for 1 h. The Normal Human Dermal Fibroblasts (NHDFs) were chosen due to their presence in the dermal layer of the skin, the random orientation of the fibres mimics the ECM of the native tissue [5]. NDHFs were seeded at 50,000 cells/well in 20 µL of Fibroblast Growth Medium 2 (FGM2) and left in the incubator for 2 h. The medium was then topped up to 1 mL and allowed to culture for 7 days (*n* = 6). After 7 days, the viability of the cells was assessed by alamarBlue™ Cell Viability Reagent (Thermo Fisher Scientific, Leicestershire, UK). The alamarBlue was added at a 10% solution in the medium in 200 µL aliquots to each well and left for 4 h, after which fluorescence was measured at 560/590 nm using a SpectraMax iD3. 

The samples’ fluorescence was compared in Origin 2020 (Academic) 64-bit using a one-way Analysis of variance (ANOVA) with a Tukey post hoc test. Results were considered significant if the *p* value < 0.05.

## 3. Experimental Results

The Taguchi design method is used to isolate the optimal conditions for PCL sponge formation and then to further assess the contributions of the polymer concentrations and humidity. These initial experimental results point also to the significance of the solution conductivity, and lead to the addition of gelatine and further experimental results that identify the PCL/gelatine sponge manufacturing conditions. We then present the results that confirm the composition of the PCL/gelatine sponges and assess their porosity, fibre diameter and hydrophilicity. We then present the results of the cytocompatibility testing.

### 3.1. The Manufacturing Conditions for PCL Sponge Formation 

**Figure A1:**
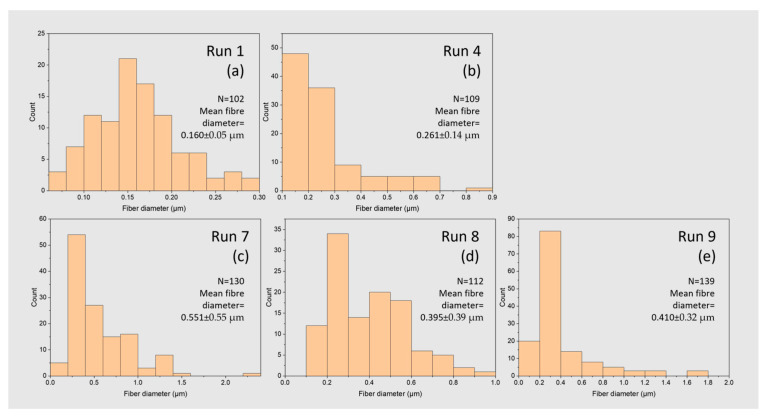
Fibre diameter histograms for initial Taguchi experiment. Refer to Table 2 in the main text.

The L_9_ table of experimental parameters, the experimental quality score and the fibre diameters are summarized in Table 2. Histograms of the fibre diameter can be found in Figure A1. All runs were completed at ambient room temperature and humidity, which, for analysis, were treated as noise at first. Humidity is not controlled at this point and therefore cannot be used as a parameter within the initial Taguchi. However, once humidity control is achieved, it can be treated as a variable in the Taguchi table, moving to an L16 orthogonal array of experiments. As will be seen later, humidity plays a significant role in reproducible sponge formation and requires control. Fibre diameter is related to the fibre’s mechanical properties (thicker = stiffer fibres) and plays a role in cell attachment and viability [31,32]. 

**Table 2:** Taguchi L_9_ matrix shows conditions, humidity, temperature, sponge production results and fibre diameter at each run. No fibre deposition resulted in the diameter being not applicable (N/A).

Humidity (%)	Temperature (°C)	Run No.	Distance (cm)	Voltage (kV)	Rate (µL/h)	*w*/*v*%	Sponge Quality Score	Fibre Diameter (µm)
**43**	23	1	10	12	500	14	2	0.160 ± 0.005
		2	15	16	1250	14	1	N/A
		3	20	20	2000	14	1	N/A
**36**	24	4	15	20	500	18	4	0.260 ± 0.015
		5	20	12	1250	18	1	N/A
		6	10	16	2000	18	1	N/A
**38**	24	7	20	16	500	22	4	0.550 ± 0.030
		8	10	20	1250	22	4	0.400 ± 0.020
		9	15	12	2000	22	3	0.410 ± 0.030

Table 2 shows that the fibre diameter roughly doubles with each step increase in *w*/*v*%, increasing from a mean fibre diameter of 0.160 ± 0.005 µm at 14 *w*/*v*% to a mean fibre diameter of 0.450 ± 0.05 µm at 22 *w*/*v*% (average of all three results at 22 *w*/*v*%). Additionally, the larger diameter fibres have higher sponge quality scores, meaning that increasing the solution concentration and thus increasing the fibre diameter does indeed improve the chance of sponge production because of the increased mechanical stiffness of the fibre. Where no fibre deposition occurred, the diameter value was not applicable (N/A).

The sponge production scores were further analysed with the ‘larger-is-better’ option to produce a response table (Table 3) for the Signal-to-Noise ratio to understand the contribution of each of the experimental parameters to sponge production [38].

The response table (Table 3) shows that concentration had the greatest impact, followed by the feed rate. However, the voltage and working distance had little impact on sponge production in the chosen ranges.

We identified the optimal conditions within the Taguchi design set in Table 2 as the concentration 22 *w*/*v*%, distance of 15 cm, feed rate 500 µL/h and voltage of 20 kV. However, the feed rate and voltage are also related to the solutions’ viscosity and conductivity, and these would require a separate investigation, beyond the scope of this work.

**Figure 1:**
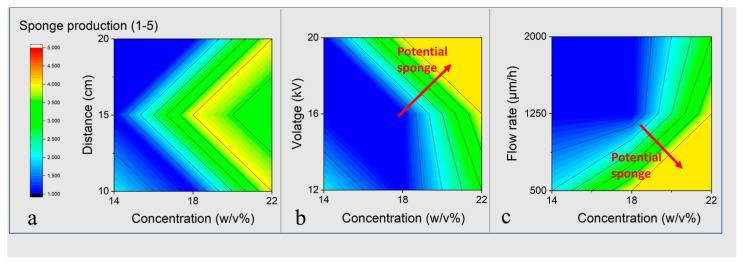
Contour plots of the PCL sponge production score against pairs of parameters. Concentration was compared to (**a**) distance, (**b**) voltage and (**c**) flow rate. No full direct sponge formation was achieved. However, this increase in quality score indicates (red arrow) that increasing concentration and voltage may lead to sponge production.

The contour plots in Figure 1 show a pair-wise analysis of parameters and their effect on sponge formation. Sponge quality score was increased with concentration; these conditions formed the basis of the experimental tests. 

In addition to the parameters set above and given the concentration playing such an important role in sponge production (Rank 1 in Table 3), it was increased towards the solubility limit (~28 *w*/*v*%). However, this proved difficult to effectively electrospin, either due to clogging or due to the very high solution viscosity, beyond the ability of a regular syringe pump to push through the thin needle. Previous research also shows that a solution to sponge formation is to change the solidification rate of the fibres (by changing humidity and/or solvent system) and to vary the conductivity (by changing solvent systems, or by adding more conductive blends to the polymer solution) [19,24,25,26]. To start with, concentration and humidity were increased independently until sponge formation occurred.

### 3.2. Preparation Parameters Effect on the Physical-Chemical Characteristics of PCL Sponge

An increased concentration reduces the fibres’ solidification time, and the increased fibre diameter allows better structural support before the fibres are fully attracted to the collector [39]. New solutions were made at 26 and 28 *w*/*v*% near the solubility limit and were spun using the optimum distance and voltage from the previous experimental set (Table 3): 15 cm and 20 kV. At ambient room conditions, no sponges formed at either concentration. Additionally, the more viscous solution required a higher feed rate for stable spinning of 10000 µL/h, but this caused the deposition of wet fibres on the collector. To improve the sponge formation, the humidity was increased for the 26 *w*/*v*% solution [26].

**Figure 2:**
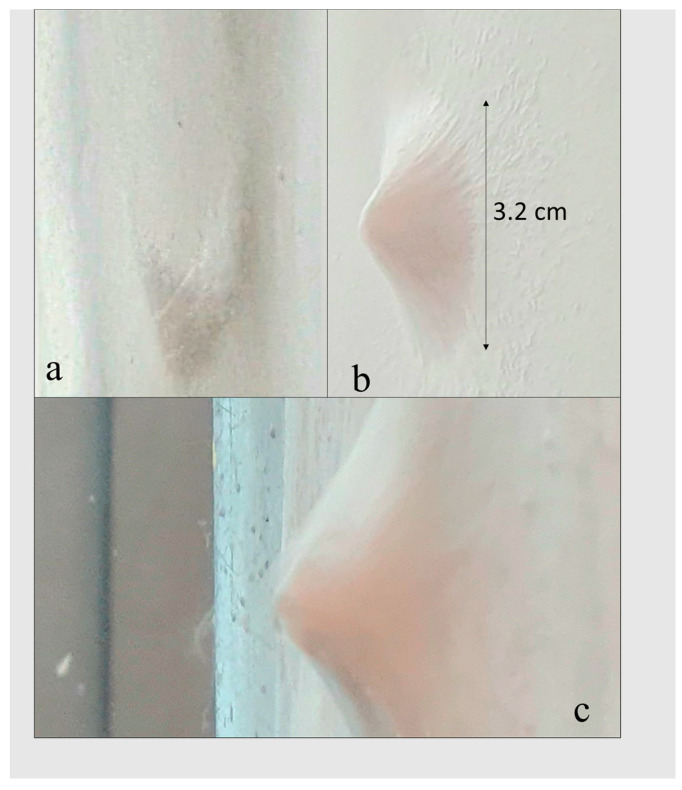
Electrospinning of 26 *w*/*v*% PCL solution at ambient and high humidity. (**a**) At ambient humidity, the fibres attempted to form a 3D structure but immediately flattened onto the collector, while (**b**) at high humidity (60%), a sponge-like structure of 3.2 cm diameter was obtained. (**c**) The sponge produced at 24 *w*/*v*% 80:20 PCL:gelatine on aluminium foil at ≥70% humidity, 15 cm, 22 kV and 1500 µL/h. The PCL/gel sponge is much fluffier.

Water’s antisolvent properties allow the elution of the solidified polymer from the acid solvents. The conditions of 26 *w*/*v*%, 15 cm, 20 kV, and 10 000 µL/h were repeated at ambient and high (50–69%) humidity. At ambient conditions (Figure 2a), a sponge-like structure attempted to form; however, the fibres were immediately attracted and collapsed flat to the collector, not forming a lofty or a three-dimensional structure. However, in high humidity conditions, a small dense sponge of 3.2 cm formed at 60% humidity (Figure 2b), proving that sponge formation is possible for a pure PCL solution. However, the sponge produced is not very practical for tissue engineering due to mass production and restriction of cell growth and differentiation [39,40]. With the previous improvements to sponge formation exhausted, an alternative method in aiding sponge formation is required by increasing the charge of the fibres.

### 3.3. The Manufacturing Conditions for PCL/Gel Sponge Formation-Increasing Conductivity 

Increasing the surface charge that the fibres can hold has several effects on sponge formation. Firstly, fibres of the same charge repel each other, increasing the space between fibres during deposition, allowing for faster volume build-up in the third dimension [9,24,25,26,40]. 

We can use a bio-compatible additive, such as gelatine, to increase conductivity. Gelatine has previously been used as an additive in the electrospinning of bio-compatible scaffolds [9,29,41] and in the formation of bio-compatible hydrogels [42]. However, to our knowledge, no PCL/gelatine sponges have been reported to date. Gelatine’s charge depends on the pH of the solution, and in the low pH of our acid solution, gelatine’s net charge would be positive [42]. 

At 22 *w*/*v*%, the 60:40 PCL:gel solution was not fully homogenous after 3 h of stirring and was difficult to electrospin due to too much dripping, with no signs of loftiness. The 80:20 solution at 24 *w*/*v*% produced loftiness but collapsed onto the collector at low ambient humidity. The 24 *w*/*v*% solution was lofty when the humidity was ≥70% on the silicone release paper but again collapsed. The silicone release paper was replaced with aluminium foil, resulting in a successful sponge formation (Figure 2c); the manufacturing conditions were ≥70% humidity, 15 cm, 24 kV and 1500 µL/h. Note that this contradicts the argument that fibres hold a charge and repel each other, as discussed earlier and in Yousefzadeh et al. (2012) [19], because the aluminium collector would facilitate fibre surface charge discharging. However, a metal collector also leads to a much stronger field at the collector surface and, therefore, facilitates the alignment of the electrospun fibres to the stronger electric field lines towards the needle, leading to loftiness, as proposed by Sun et al. (2012) [26]. To ensure reproducibility, the sponge was formed on five separate occasions.

### 3.4. PCL/Gelatine Sponge Characterisation—Fibre Morphology

**Figure 3:**
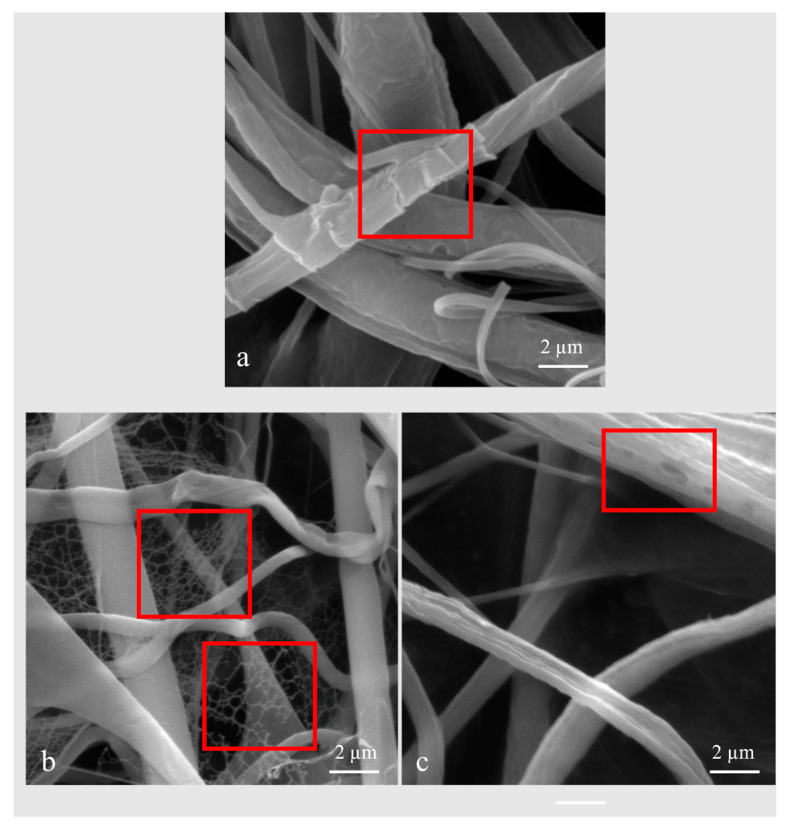
(**a**) PCL sponge 26 *w*/*v*%, 15 cm, 20 kV, and 10,000 µL/h, 26 °C, 60% humidity, SEM image showing roughness of fibres. (**b**) SEM fibres 80:20 PCL/gel 24 *w*/*v*% on foil, 24 °C, 31% RH, 1500 µL/h, 15 cm, 22 kV showing webbing and (**c**) 80:20 PCL/gel sponge 24 *w*/*v*%, 78% RH, 30 °C, 1500 µL/h, 15 cm, 22 kV showing loss of webbing and fibre porosity. Images were taken with a field view of 15 µm.

**Figure A2:**
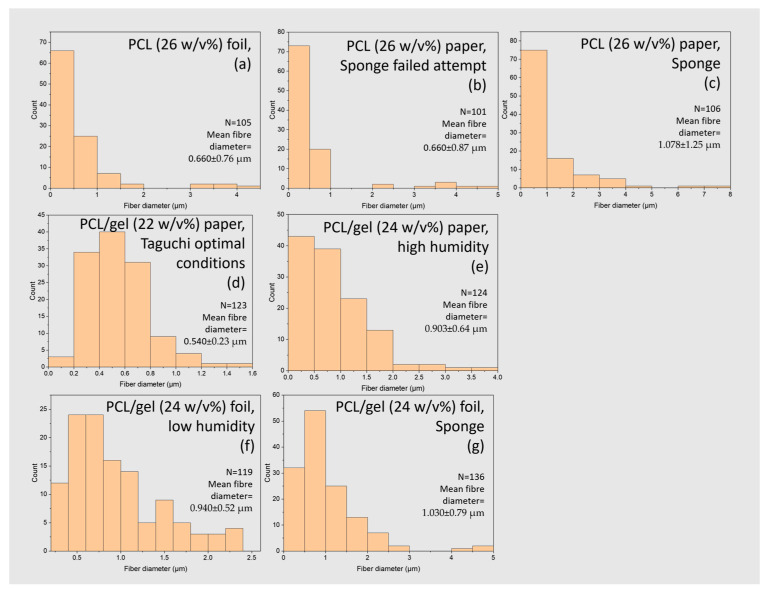
Fibre diameter histograms for condition adjustments, including PCL sponge and PCL/gel sponge.

The pure PCL sponge had a mean fibre diameter of 1.07 ± 0.12 µm, and the PCL/gelatine sponge had a similar mean fibre diameter of 1.03 ± 0.07 µm (Figure 3). Histograms for sponge fibre diameters can be found in Figure A2. Additionally, the PCL/gel sponge fibres had a slightly larger diameter than their comparative fibres at low humidity of 0.93 ± 0.05 µm; although the difference is marginal, it could be due to the absorption of the water molecules by gelatine, causing the fibres to swell.

The pure PCL sponge showed signs of fibre roughness (Figure 3a), which could be caused by decreased electrostatic charge dissipation [29]; this could be verified with a careful analysis of the morphology of the fibres as a function of the conductive nature of the substrate but is beyond the scope of this paper. The PCL/gelatine fibres at low ambient humidity showed a thin nanofibre webbing (Figure 3b), and the large diameter fibres disappeared at high humidity (Figure 3c). This could indicate a phase separation of the solution but would require further investigation. At high humidity (Figure 3c), the sponge fibres also show some porosity, but this could be due to vapour-induced phase separation, as suggested by Nezarati et al. [43]. This porosity is advantageous for improving cell attachment migration and reducing cell aggregation [44]. 

### 3.5. PCL/Gelatine Sponge Characterisation—Fibre Composition by FTIR

**Figure 4:**
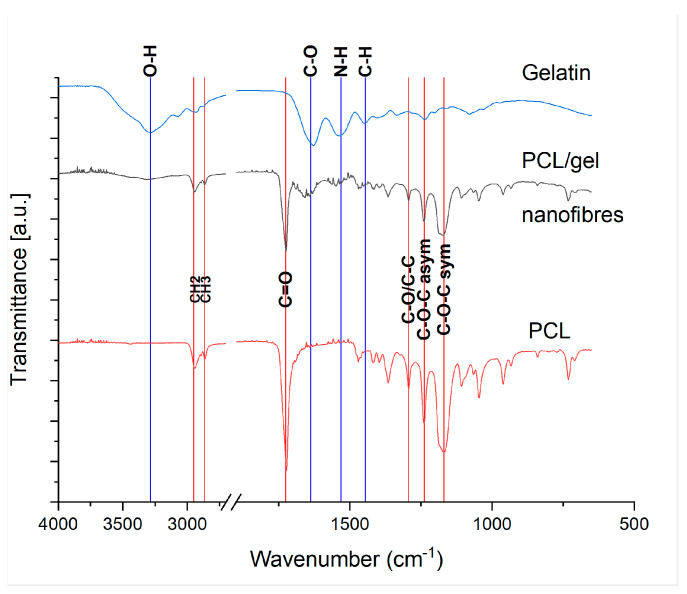
FTIR with a resolution of 2 cm^−1^ of pure gelatine (blue), pure PCL (red) and the PCL/gel (black) nanofibers show that the PCL/gelatine nanofibers contain both PCL and gelatine. Gelatine-specific peaks are: O-H group at 3287 cm^−1^, C-O group at 1638, N-H group at 1532, and the C-H group at 1446. The PCL-specific peaks are: CH_2_ at 2949, CH_3_ at 2868, the C=O at 1726, and the carbon and oxygen groups between 1294 and 1169.

Figure 4 confirms the presence of both PCL and gelatine in the fibres of the sponge formed at 24 *w*/*v*% 80:20 PCL:gelatine with the gel O-H group at 3287 cm^−1^, C-O group at 1638 cm^−1^, N-H group at 1532 cm^−1^ and the C-H group at 1446 cm^−1^. The PCL groups for CH_2_ at 2949 cm^−1^, CH_3_ at 2868 cm^−1^, the C=O at 1726 cm^−1^ and the carbon and oxygen groups between 1294 cm^−1^ and 1169 cm^−1^. The PCL and gelatine used were the pure forms from Section 2.1.

### 3.6. PCL/Gelatine Sponge Characterisation—Morphology and Hydrophilicity

The PCL sponge had a porosity of 88.8 ± 4% and a density of 0.129 ± 0.01 g/cm^3^, whilst the PCL/gel had higher porosity (96.4 ± 1%) and ~3x lower density of 0.043 ± 0.001 g/cm^3^. The experiments were replicated three times with consistent outcomes.

**Figure 5:**
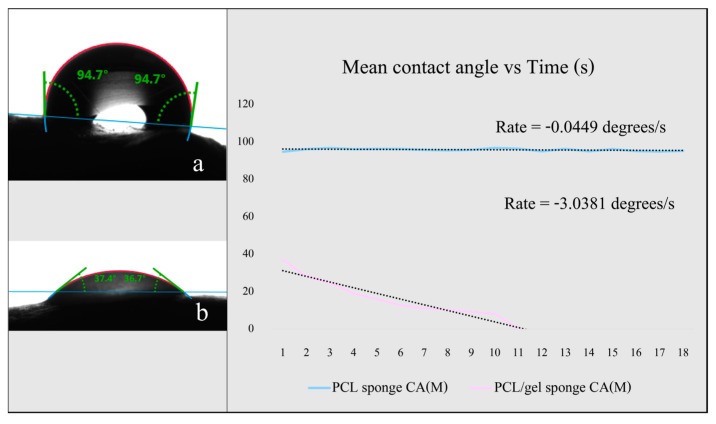
(**a**) The initial contact angle for PCL nanofibers shows a hydrophobic nature, whilst (**b**) the initial contact angle for PCL/gelatine shows a hydrophilic nature. Water does not absorb easily in the PCL sponge, whilst the PCL/gel sponge fully absorbs it within seconds.

The hydrophilicity of the sponges was assessed by measuring the initial mean contact angle (Figure 5); the lower the contact angle, the higher the hydrophilicity. The initial mean contact angle for the PCL sponge (Figure 5b) was 94.7°, and the rate of decrease was 0.045°/s while the PCL/gel sponge (Figure 5a) showed a contact angle of 37.05° and the rate of decrease was 3.038°/s. The PCL/gel sponge was much more hydrophilic, with much higher water absorption, and did not require plasma treatment for cell culture. 

### 3.7. 3D PCL/Gel Sponges Support Superior Growth of Dermal Fibroblasts

**Figure 6:**
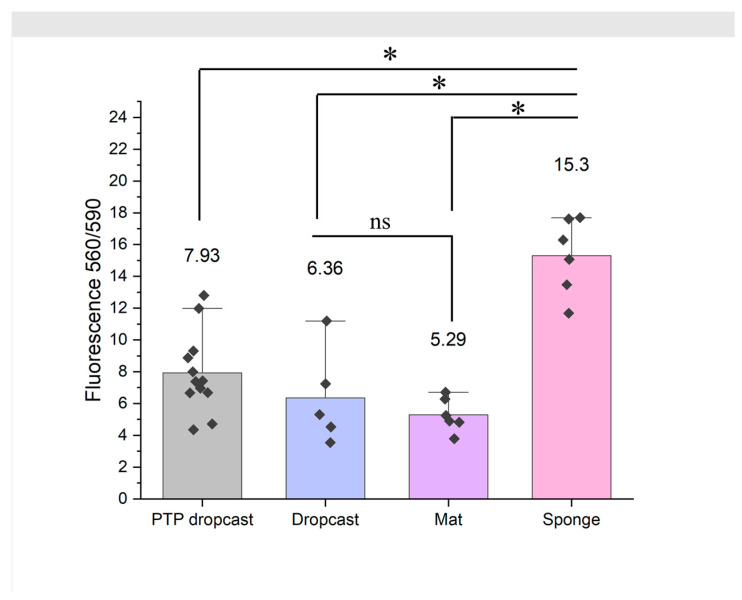
NHDF comparative cell viability study using a 4-h alamarBlue fluorescence assessment shows that the PCL/gel sponge is twice as viable compared to the respective mat and film of PCL/gel and the positive control of assessment. A one-way ANOVA confirms the sponge result is significant (* = significant difference, ns = not significant difference). Plasma treated polystyrene (PTP) was used as a comparison of current cell culture techniques on this material. Each point (**⬩**) shows the fluorescence results for each culture replicate the top of the bar gives the mean fluorescence value, and the error bars give the standard deviation (SD).

Figure 6 shows that the PCL/gel sponge supported the attachment and growth of almost double the number of NHDF cells, as compared to the 2D PCL/gel nanofibre mat, the 2D PCL/gel dropcast control and the 2D PTP dropcast after 7 days. Although this is a brief study that requires further confirmation with confocal microscopy (to confirm cell presence and distribution within the 3D volume), the sponge shows increased cell growth. No difference in cell growth was observed between the PTP control for the PCL/gel dropcast control (*p* = 0.60881) and the PCL/gel mat control (*p* = 0.14483). The PCL/gel sponge had significantly greater cell growth than the PTP, PCL/gel dropcast and mat (*p* = 9.94547 × 10^−6^, *p* = 9.73475 × 10^−6^ and 7.12219 × 10^−7^, respectively), where *p* is significant <0.05. This indicates that the NHDFs prefer the 3D nanofibre environment.

## 4. Discussion

The Taguchi method of design is generally used to identify optimal manufacturing envelopes over which the quality of the product is consistently over a certain level. It is not best at local minima and maxima [36] but it can indicate directions of change. In our case, it allowed for both an initial isolation of optimal parameter conditions and identified that the PCL solution concentration could have the highest contribution to the production of sponges. At the same time, increasing the concentration increases the fibre diameters; increasing the fibre diameter can be beneficial [45] but does come at the cost of a reduced surface area. By increasing the concentration towards the solubility limit improved the sponge production quality score for both the PCL and PCL/gel solutions. However, high concentrations made electrospinning impractical, requiring very stiff syringes and often resulting electrospraying. As a result, we increased humidity (>60%) to accelerate solidification [24,25] where water acts as an antisolvent [46]. This resulted in the successful manufacture of PCL sponges that were relatively dense at 0.129 g/cm^3^ and had a relatively low porosity of 88.8%. Consequently, we modified the viscosity by adding gelatine, which also increases the conductivity of the spinning solution [42]. In the two existing theories explaining sponge formation, electrospun fibres form into sponges, either because they align with the electrostatic field lines between the needle and the substrate [26], or because they carry charge that dissipates slowly upon landing on the substrate such that fibres repel each other electrostatically into a sponge structure [19]. When we used an insulating substrate (silicone coated paper), we produced PCL sponges; however, this did not repeat with PCL/gel sponges. Changing to a conducting substrate increased the field strength between the needle and the collector and resulted in the successful manufacture of PCL/gel sponges. These sponges then proved to be twice as viable for NHDFs after 7 days when compared to controls of PCL/gel 2D nanofibers, dropcast films and PTP dropcast films. 

One particular challenge here is how a meaningful comparison can be made between a 2D and a 3D structure. The same cross-sectional area was chosen by the well diameters in the well plate; however, one could consider the surface area available for cell attachment instead (which would require some form of surface area measurement, e.g., gas adsorption) or simply the weight of substrate used. In this case, we decided to use the well cross-section, which is the common denominator in cell growth techniques. Future work will explore methodologies that can consider the challenge of comparing 2D vs 3D architectures and growth models.

## 5. Conclusions

This study investigated the effects of concentration, humidity and conductivity on the production of PCL sponges using the Taguchi design method. The results showed that all three factors significantly impacted the sponge formation. The sponges require a high-concentration solution and high humidity in all conditions. A pure PCL sponge will form at 26 *w*/*v*%, 15 cm, 20 kV and 10 000 µL/h at 60 *w*/*v*%. However, it is relatively dense at 0.129 g/cm^3^ with a comparatively low porosity of 88.8%. The optimal conditions for producing a PCL sponge with high porosity and hydrophilicity required a 20% gelatine solution to increase conductivity. The sponge formed at 24 *w*/*v*% concentration, ≥70% humidity, 15 cm distance, 22 kV voltage and 1500 µL/h feed rate. The PCL/gel sponge produced under these conditions had a density of 0.04 g/cm^3^ and a porosity of 96.4%. The hydrophilicity of these sponges was then compared with the PCL/gel sponge, having a significantly greater hydrophilicity with a mean initial contact angle of 37.05° compared to 94.7° of the pure PCL sponge. The three-dimensional sponge had improved cytocompatibility, as evidenced by the significantly higher (~2X) NHDF cell viability compared to the control mats and films. Additionally, the sponge had a significantly greater viability than the dropcast TCP positive control. These results suggest that PCL/gel sponges have great potential for tissue regeneration applications due to their biocompatibility and biodegradability.

## Figures and Tables

**Table 1:** Sponge production qualitative key. Observations during fibre deposition are assigned an empirical score.

Sponge Quality Score	Observation
1	No electrospinning or mostly dripping
2	Electrospinning with some dripping or unstable Taylor cone
3	Stable electrospinning, continuous fibre formation
4	Some fibres do not deposit flat
5	Sponge production

**Table 3:** Response table for signal-to-noise ratios (larger-is-better). Delta is the highest-to-lowest SNR difference and indicates the parameter with the highest impact and is used to rank the parameters in order of their contribution to sponge production control. Note that working distance and voltage are related through field strength and may not be independent.

Level	Concentration SNR	Feed Rate SNR	Working Distance SNR	Voltage SNR
1	2	10	6	5
2	4	4	7	4
3	11	3	4	8
Delta	9	7	3	4
**Rank**	**1**	**2**	**4**	**3**

## Data Availability

These data are available upon request. Unsterilised PCL/gel sponge samples can be provided upon request for ethical research.
